# A Qualitative Study of the Subjective Appraisal of Recovery Among People with Lived Experience of Schizophrenia in Poland

**DOI:** 10.1007/s11126-016-9459-6

**Published:** 2016-07-25

**Authors:** Izabela Nowak, Justyna Waszkiewicz, Piotr Świtaj, Marlena Sokół-Szawłowska, Marta Anczewska

**Affiliations:** First Department of Psychiatry, Institute of Psychiatry and Neurology, Sobieskiego 9, 02-957 Warsaw, Poland

**Keywords:** Schizophrenia, Recovery, Service user experience, Qualitative research, Interventions

## Abstract

The objective of this study was to explore definitions of recovery among Polish service users with lived experience of schizophrenia and to hear their recommendations regarding elements that should be considered in the planning of a recovery oriented psychosocial intervention. Four semi-structured focus groups were conducted in the Institute of Psychiatry and Neurology in Warsaw, Poland. A total of 28 service users’ narratives were examined using the inductive thematic analysis approach. Five main recovery themes emerged from the combined users accounts, listed in order of frequency: psychological dimension of recovery, relationships with others, wellness strategies, clinical understanding of recovery and support systems. Service user recommendations referred to the above identified recovery themes as well as indications that the intervention should be flexible, individualized, and facilitative of personal growth. The findings indicate that for service users with lived experience of schizophrenia in Poland it is culturally feasible to embrace the person-oriented approach to practice and develop a recovery-oriented psychosocial intervention emphasizing psychological domains of recovery such as positive identity, personal strengths, or meaning and purpose in life alongside the other relevant recovery dimensions. Actions regarding the system level of change are also required.

## Introduction

Schizophrenia is a psychotic disorder that impacts upon all major areas of life. Using the framework of the International Classification of Functioning, Disability and Health (ICF) [1] the scope of psychosocial difficulties experienced by people diagnosed with schizophrenia goes beyond impairments of mental functions (schizophrenia symptoms) and refers to several activities and participation domains for example relationships with others, employment as well as environmental factors such as availability of treatment, country-specific systems and policies, or stigmatizing societal attitudes [2, 3]. There has been a growing need to meet the challenge of disability experienced by people with lived experience of schizophrenia from a perspective that extends symptomatic improvement.

A crucial part of this broader perspective is the recovery paradigm, which de-emphasizes the notion about clinical remission, and instead embraces the idea of “living a satisfying, hopeful, and contributing life even with limitations caused by illness” [4]. Service users have identified some key components of recovery, such as regaining hope, empowerment, social connection, having meaning and purpose in life, transformation of identity, re-assuming responsibility and control, managing symptoms and combating stigma [5]. The conceptual framework for personal recovery developed by Leamy et al. [6] in the process of a systematic review consists of similar aspects, specifically: connectedness, hope and optimism about the future, identity, meaning and purpose, and empowerment (giving the acronym CHIME). There are also typologies of recovery dimensions that in addition to the subjective or existential aspects include broader views of the concept such as human rights, a positive culture of healing in service provision, recovery-oriented services [7], and clinical, functional, physical or social aspects [8].

Personal recovery has been increasingly prominent over the last years and currently underpins mental health policy in many countries [9]. Along with its growing importance in policy, cultural differences in the values fundamental to the concept of recovery have emerged [10]. For instance, a number of differences regarding the concept of recovery have been found among people from minority communities [11]. These referred to the role of spirituality and religion; culture-specific traditional healing practices; individualistic versus collectivist meaning and experience of recovery; and additional stigma and discrimination faced by individuals and their families or barriers at the level of the mental health system, including perceptions of institutional racism. This supports the notion that the primary aim of recovery-oriented care is to offer people with serious mental illness a range of effective and culturally responsive interventions [12].

In Poland the predominant model of care remains hospital centered with coexisting ambulatory care, without regionalized service provision. The shift to the community-based facilities and the recovery orientation is not progressing as fast as it should due to organizational and financial issues [13]. In the early phases of local service transformation, exploration of service users’ perspective on recovery is necessary for the development of interventions facilitating the process of personal recovery. Therefore the aim of our qualitative study is to identify the meaning of recovery among Polish service users diagnosed with schizophrenia and to hear their views regarding recommendations for a meaningful psychosocial intervention.

## Materials and Methods

A focus group method was used to investigate the opinions of people with lived experience of schizophrenia regarding the recovery concept and recovery elements to be considered in the planning of a recovery oriented intervention. This methodology has become increasingly useful in informing mental health policymakers about recovery conceptualizations [14] and can be applied to exploratory studies [15] and the development of new programs [16]. A focus group interview was developed by authors, containing questions about the meaning of recovery and personal recovery elements to be addressed in a psychosocial intervention. To facilitate the group discussion and to clarify the service users’ narratives, a range of additional questions were asked as specified by Kvale [17].

## Participants

A purposive sample of participants was selected from four different settings: in-patient ward, rehabilitation day unit, day center and community team at the Institute of Psychiatry and Neurology in Warsaw. The variety of settings ensured diversity in service users’ responses [18]. Participants were recruited to the study if they met the following criteria: schizophrenia diagnosis (F20, the International Classification of Diseases, ICD-10), without mental disorder comorbidity, age ≥18 years, and cognitive capacity to participate in a focus group. Participants were clinically stable at the time of focus group that is with unwavering mental status and fixed treatment. Diagnoses were made by the psychiatrist responsible for treatment and care in the settings under the study. The sample size was estimated according to Kitzinger and Barbour [19] indication about the number of participants in each focus group ranging from 3 or 4 individuals and up to 12 persons. Following Brown [20], we also estimated that with data from four groups, saturation would be reached.

## Data Collection

Ethical approval was granted by the Bioethics Committee at the Institute of Psychiatry and Neurology. The objectives and procedure of the study were presented during community meetings taking place in the in-patient ward, rehabilitation day unit, and day center, where informative posters were also displayed. People interested in the study were screened based on the inclusion criteria. Service users from the community team, who met the inclusion criteria, were contacted by phone and asked whether they would like to participate in the focus group. All service users signed their informed consent prior to the commencement of the study. Participants were reminded about their right to withdraw and the strict confidentiality regarding personal data at the beginning of each focus group. They also agreed to maintain the confidentiality of the discussion, as well as being encouraged to express their opinions even if they felt it had been previously stated. The focus groups lasted 60–90 min and were led by two psychologists, a moderator and co-moderator who were not involved in participants’ care (IN, JW). The moderator leaded the meetings by introducing the topic, asking the targeted questions and moderating the discussion. The co-moderator facilitated with the discussion moderation and took notes. Each focus group took place in a designated room, reserved for the meeting and was audio-recorded. Upon completion of the focus groups, participants were asked to complete a demographic questionnaire which included information regarding age, gender, education, employment, civil status, and duration of treatment.

## Analyses

All interviews were transcribed verbatim and anonymized. An inductive thematic analysis procedure was used to analyze the data [21]. First, two independent coders familiarized themselves with the transcripts (IN, JW) by reading them and writing down first ideas. This was followed by initial semantic-level coding of the data with regards to the interview questions. Afterwards, the researchers compared and re-negotiated the coding results; initial names for themes were proposed. Subsequently, systematic reviewing of themes was undertaken by one researcher (IN). This involved themes refinement regarding their name, definition, exhaustion and relation to the coded extracts and the entire data set. An extract of a narrative could be included in more than one theme. At the end of this process the entire data set was again evaluated and reviewed by the two researchers (IN, JW). This involved discussions and re-negotiation of themes and codes within the themes to ensure their coherence and validity. As a result of this process, definitions and names for codes were generated which were collated into five key themes.

## Results

The final sample consisted of 28 participants: 9 service users from the inpatient ward, 8 from the rehabilitation day unit, 4 from the day center, and 7 from the community team. The mean age was 43.78 years [standard deviation (SD) = 13.80], and the mean years of using mental health services was 15.08 (SD = 10.20). The majority of the participants were men (56.60 %), single (67.90 %), with vocational education (53.60 %), and unemployed—receiving disability benefits or pension (74.10 %). Nearly one-third (28.60 %) of the participants had a university degree.

Five main recovery themes were derived from the combined service users’ narratives listed in order of frequency: psychological dimension of recovery, relationships with others, wellness strategies, clinical understanding of recovery and support systems (Table 1).

**Table 1:** Main themes of the recovery concept

Psychological recovery (n = 16)	Relationships with others (n = 15)	Wellness strategies (n = 12)	Clinical understanding of recovery (n = 9)	Support systems (n = 5)
Identity transformation	Contact with people	Being active	A lack of symptoms	Livelihood
Meaning and purpose	Communication	Leading a healthy lifestyle	Return to a former state of health	Psychiatric mental healthcare
Emotional well-being		Coping with symptoms	Attitude towards medication	
Recovery being a process			A lack of difficulties related to illness	

*n* number of participants

In relation to recommendations for a psychosocial intervention, people referred to all identified recovery themes, expected structure, and favorable method of implementation.

### The Concept of Personal Recovery

*Theme 1* Psychological dimension of recovery. This dimension of recovery was the most frequently endorsed recovery theme. It was reported by a total of 16 focus group participants and consisted of identity transformation, finding a meaning and purpose, emotional well-being and recovery being a journey. Identity transformation referred to a number of processes, which were categorized into the development of the motivation to change, illness awareness and personal growth. Personal growth was the most frequently reported aspect of identity transformation, emphasizing the importance of developing positive self-identity with narratives referring to the acceptance of illness and oneself, the development of self-esteem and self-empowerment, psychological distancing, or stigma management. This reflects the following service user quotation: “Recovery is about not stigmatizing oneself … seeing that I am not the illness itself … [it is] also seeing oneself as a worthwhile person … because I survived this illness … so discovering self-value with self-esteem that relates to these experiences.” Participants also reported that finding the meaning and establishing goals in life was another factor relevant to recovery: “discovering the meaning of life … the purpose of life … leading the life according to good values … discovering the meaning and discovering the values.” The need of discovering the meaning of illness or spiritual development was also a common narrative, e.g. “It depends on one’s worldview but I think, as many of you said, faith and prayer helps a lot.” Goals were described in the context of establishing values, vocational reintegration or leading a responsible life free of debts, problems with the law and correcting problematic behaviors. The subcategory of well-being was reflected by narratives about feeling good with oneself, inner peace and achieving life equilibrium. One of the focus group participants explained that emotional well-being was paramount to medication: “… human well-being is so important … that the person feels good. Medication [can help] but the person has to feel good and this is the most important and this is recovery.” Life equilibrium was attributed to a peaceful life, moderation, having contact with nature, and a life-work balance. Recovery was also described as a long-term process in which the challenges of illness change. This process is not focused on “being cured” but on overcoming illness difficulties as described: “I have been ill for the last 16 years … [at the beginning] I [hoped] that I would be completely cured… but now I think it’s about overcoming the illness … perhaps all my life I will live with it … [I need] to accept it somehow ….”

*Theme 2* Relationships with others. Another theme of recovery contained narratives related to relationships with others. A total of 15 participants reported relationship experiences, which were grouped around contacts with people and communication. Some participants expressed that having contact with other people, as well as engaging in social activities were both essential to recovery. Developmental aspects of interpersonal contacts also emerged which was interrelated with personal growth: “Working on what is difficult for me helped me to initiate relations with people … I began to understand more myself … a greater empathy to people was developed, openness, trust.” Regarding communication, some participants underlined its importance in establishing contacts with people. These referred to the importance of talking with people, managing conflicts or the increased sense of connectedness as a result of communicating with others. The following account emphasizes the importance of sharing the experience of illness in contributing to its better understanding: “Well, listening to others… helps in understanding that things like this happen in this illness.”

*Theme 3* Wellness strategies. A total of 12 participants reported a range of wellness strategies contributing to recovery, which were categorized into the following subthemes: being active, leading a healthy lifestyle and coping with symptoms. Being active was attributed to motivational aspects as well as leading an active life. Motivation was driven internally and externally. For instance one of the participants underlined the role of self-motivation in dealing with inactivity: “Overcoming inactivity, forcing oneself to be active … simply creating intentions to do something despite difficulties. This motivation is important.” However, another participant underlined the value of participation in activities at the hospital as mobilizing: “Recovery for me [is] maybe when I am a bit more active. Because before I felt like doing nothing, nothing interested me, and I come here and this helps me a bit … this mobilizes me.” Activities described in this subtheme considered daily commitments at the hospital or recreation and activities outside of the hospital, such as going to the cinema, theatre, library, engaging in social relationships, and daily life activities (e.g. cooking, walking a dog or sports). A number of accounts emerged that were categorized as healthy life habits. These were described in terms of eating regularly, taking medication, exercising, praying, developing healthy interests and hobbies, looking after oneself, regular visits to a doctor and following medical advice. Coping with symptoms was mostly attributed to coping with hearing voices and having control over them as well as overcoming fears and having emotional self-control, for instance “For me the element of recovery is … I control the voices, that the voice don’t take control over me … I can separate it … I am aware it is an element of illness.”

*Theme 4* Clinical understanding of recovery. The clinical understanding of recovery was reported by a total of 9 focus group participants. Narratives reflecting this theme included a lack of symptoms, return to a former state of health, attitude towards medication, and a lack of difficulties related to illness. One of the most debated aspects of this pertained to medication. Most of the participants underlined the necessity of taking medication, while emphasizing that pharmacotherapy had to be carefully managed. One of the participants stressed that the right kind of medication is crucial in recovery: “I would insist however … it is important the prayer, eating well and all that [are important], but in this illness well-prescribed medication is 80 % of success.” However, another participant clarified that medication does not prevent relapse: “I take medication … and despite taking medication I relapse, completely out of nowhere … I take medication regularly, at the same hour, I care so much about being healthy and functioning normally, but despite this, the illness comes back.”

*Theme 5* Support systems. Five participants reported that access to supportive systems fosters recovery. This was attributed to a secure livelihood and satisfactory psychiatric mental healthcare. Matters of livelihood considered difficulties in navigating and obtaining information within the support system but also stressed the importance of the security of accommodation, food, and economic stability in recovery as depicted in the following quotation: “Livelihood matters are important, to have something to eat and security of accommodation … financial stability is important as well.” The subcategory of psychiatric mental healthcare referred to the availability of mental health services and the quality of relationships with mental health professionals. The need of treatment from the outside of the hospital emerged: “… as it is known I cannot go to hospital all my life, to be sick in a hospital … but a doctor that comes to you privately and will advise on medication or something.” Participants also described a number of experiences referring to the quality of contact with mental healthcare staff. These included accounts about the openness of contact, acceptance, partnership, and friendliness of these relationships. One person underlined the role of partnership in managing medication: “If I tell him, that I react badly to this drug then he will decrease the dose and prescribe another drug, or we will just talk and he will suggest something …a good doctor with whom you can talk.”

### Intervention Recommendations

The exploratory focus group study revealed a number of recommendations within each recovery theme. With regards to the psychological dimension of recovery participants’ recommendations covered the majority of identified recovery elements, that is transformation of identity, finding the meaning and purpose in life and achieving life-balance with the aspect of positive identity development being the most widely supported. The theme relationships with others reflected the need of communication and having contact with people; however, the importance of other people involvement in the process of recovery also emerged, for instance: “When I get worse I need a kind of … friend or someone from family who will visit me in the hospital.” As to wellness strategies participants recommended living outside the hospital, fulfilling daily activities and managing symptoms; however, symptoms management such as development of criticism towards symptoms was the most common narrative, for example: “… when I relapse I need someone … to tell me that what I think does not exist.” Regarding support systems participants underlined the need of information provision about available financial support when feeling ill as well as having a good quality and regular contact with mental healthcare staff, for example: “Sessions with a psychologist … once every 2 or 3 weeks, sometimes once a week…it gives me a sense of security.” Lastly the need of continuation with well managed medication was underlined, as in the following example: “Now that I have well-prescribed medication I can balance everything. I have time for prayer, eating, and earning some money.” In addition to the identified recovery themes participants suggested that the intervention should be individualized in the sense of considering individual capabilities, pace, and not being imposed in any way. “It has to be adjusted to the specific phase of recovery … as it can be hurtful … if someone is too ill [they]cannot participate in this intervention.” There were also views indicating that treatment should be conceptualized as a personal development process and that it would be unconstructive if it reinforced peoples’ feeling about being ill: “… a kind of developmental coaching … showing people possibilities [in life] … if you fear something you don’t have to focus on the fear and take the pill …” or “It depends on the people that surrounds us … the belief that we are not normal … it would be a negative intervention.”

## Data Saturation

Data saturation is typically observed when no new information or themes emerge from the data [22]. In our study, the third and the fourth group described the same themes, and categories within themes, as the two previous ones. We therefore view our data as “rich, full, and complete” [23].

## Discussion

The purpose of our study was to explore the meaning of recovery among Polish service users with lived experience of schizophrenia and to receive their recommendations regarding the development of a psychosocial intervention. Various aspects of recovery emerged including: the psychological dimension of recovery, relationships with others, wellness strategies, clinical understanding of recovery, and support systems. Participants expressed a number of recommendations referring to the all identified themes as well as its expected structure, and favorable method of implementation.

The emerging recovery aspects are broad, encompassing a wide range of components relating to individual, social, functional, clinical, and environmental dimensions [6–8]. Overall, this may indicate that for participants recovery is a multidimensional concept and that the experience of psychological recovery, which was the most widely supported, happens in the context of existing relationships with others, utilization of strategies supporting health, symptomatic improvement, or a broader system support. This may also mean that for service users who took part in the focus group, clinical remission was not central to recovery; however, subjective recovery was neither a standalone experience.

Psychological recovery involved developing positive self-identity, finding a meaning and purpose, emotional well-being, or recovery being a process. These recovery aspects resemble some of the SAMHSA National Consensus Statement recovery aspects [24], the CHIME framework [6], or the strength model [25] where the focus is placed on developing people’s abilities and resources. Additionally, participants underlined the role of religion and spiritual practices as supporting ones’ recovery. Given its importance for Polish service users it may indicate that services should be sensitive to people’s spiritual needs and perhaps initiatives aiming to foster partnerships between mental health practitioners and religious groups should be launched [26].

The greatest support given to the theme of psychological recovery and relationships with others reflects another culturally relevant factor referring to the dimension of individualism and collectivism. Relationships with others revealed participants’ need of contact and communication with other people which is consistent with the findings of Hartley et al. [27] about social withdrawal, loneliness, problems with relationships and interpersonal skills being a common experience among service users’ living with schizophrenia. However, from the perspective of individualism and collectivism it may also indicate that psychological recovery and relationships with others might coexist with each other as the experience of recovery involves an ongoing process of psychological and social negotiation between modes of autonomy and relatedness [10]. This is in line with Kwiatkowska [28] indication that Polish culture is rather heterogenic regarding the dimension of individualism and collectivism, therefore in the socialization process cooperation with others as well as looking after ones’ personal needs are both important.

Lesser focus placed on clinical remission in our study de-emphasizes the view of symptomatic improvement being paramount to recovery; however it also indicates that personal as well as clinical recovery aspects support one another [29] and that clinical recovery might be influenced by other recovery dimensions [8]. For instance studies evaluating the effectiveness of recovery-oriented cognitive behavioral therapy (CBT) [30] or “third wave” CBT [31], found improvements in schizophrenia psychopathology despite that symptomatic reduction was not the primary focus of these interventions. Furthermore, it would be beneficial to explore the meaning of recovery among Polish mental healthcare staff to see whether they agree with users’ views of recovery since it seems to be of paramount importance in terms of therapeutic engagement and treatment effectiveness. Aspects of clinical recovery in our study mostly referred to the alleviation of symptoms and difficulties related to illness as well as taking well prescribed medication. Coping with symptoms also emerged; however we categorized it together with other wellness strategies, since in our view it resembles the idea of self-management through the development of ability to look after oneself by utilization of strategies promoting health [32].

The theme of support systems referred to the need for access to friendly mental health services, as well as information about available support, assistance in navigating the system, and secured livelihood. In our view it reflects the local context of mental health care as well as social security system. In Poland institutionalized care remains the dominant type of mental healthcare, accessibility to community-based care is hindered and transformation into recovery-oriented care is progressing slowly [13, 33]. Furthermore mental healthcare services within the social assistance sector are poorly integrated, which points to reduced availability of services assisting people with lived experience of schizophrenia to get back to work or school. Although these aspects go beyond the scope of a single psychosocial intervention, they are indeed relevant in the context of support systems transformation.

Overall, users’ recommendations regarding a psychosocial intervention referred to the all identified themes; however the participants also expected a flexible, individualized intervention, as in person-centered planning [34], that would accommodate individual capabilities and pace, and adapt to the phase of recovery. For example, during an acute psychotic crisis, a person’s “power” may be impaired to the extent that reduction of distress and the burden of symptoms is the primary focus of professional action. Regaining “power” for self-determination, optimism, finding new opportunities as well as establishing meaningful goals might be a focus in the subsequent stage of the intervention.

Following participants’ recommendations we presume that it would be culturally feasible to develop actively engaging and individually tailored intervention, which focuses on mobilizing users’ strengths, resources and skills, emphasizes that recovery goes beyond symptomatic reduction, facilitates the process of finding the meaning and purpose in life, enables the self-management of physical and mental health, and fosters the development of relationships with others. Following participants’ suggestions changes are also required at the level of support systems. These may refer to the development of user friendly services that offer a comprehensive support in times of crisis including not only medical help but also information about available means of support. Following the crisis availability of good quality services in peoples’ local communities is called for as well as greater security regarding livelihood matters.

Results of our exploratory study have to be interpreted in the light of several limitations. Despite the recruitment of participants’ from the four different mental health settings and data saturation, our results cannot be generalized to the entire population of people with lived experience of schizophrenia in Poland. Further research with a representative sample of service users’ is needed to explore the meaning of recovery and recommendations for a feasible intervention. A follow up meeting involving the participants of the focus groups validating the themes derived from the discourse is also recommended as well as running individual interviews to enrich the data. It is worth noting that according to Söderström [35], our results could be influenced by social desirability bias.

## Conclusions

Our results indicate that among people with lived experience of schizophrenia who took part in the study, psychological recovery lies at the heart of the concepts; however other factors such as relationships with others, symptomatic improvement, wellness strategies and systems support are also relevant to people’s recovery. The recommendations referred to the identified recovery themes but also called for the intervention to be flexible, individualized, not focusing on illness but emphasizing people’s potential for growth. Therefore, we assume that for service users’ with lived experience of schizophrenia in Poland it is culturally feasible to implement the person-oriented approach to practice and develop an intervention highlighting the psychological aspects of recovery such as positive identity, people’s strengths or meaning and purpose in life along with addressing other identified recovery dimensions. Our results also call for actions regarding the system level of change.

## References

[1] World Health Organization (2001). International Classification of Functioning, Disability and Health-ICF.

[2] Świtaj P, Anczewska M, Chrostek A, Sabariego C, Cieza A, Bickenbach J (2012). Disability and schizophrenia: A systematic review of experienced psychosocial difficulties. BMC Psychiatry.

[3] ICF Research Branch. Brief ICF Core Set for Schizophrenia. http://www.icf-research-499branch.org/download/send/9-mentalhealth/255-brief-icf-core-set-for-schizophrenia. Accessed 15 Feb 2016.

[4] Anthony WA (1993). Recovery from mental illness: The guiding vision of the mental health service system in the 1990s. Psychosocial Rehabilitation Journal.

[5] Schrank B, Slade M (2003). Recovery in psychiatry. Psychiatric Bulletin.

[6] Leamy M, Bird V, Le Boutillier C, Williams J, Slade M (2011). Conceptual framework for personal recovery in mental health: Systematic review and narrative synthesis. The British Journal of Psychiatry.

[7] Jacobson N, Greenley D (2001). What is recovery? A conceptual model and explication. Psychiatric Services.

[8] Whitley R, Drake RE (2010). Recovery: A dimensional approach. Psychiatric Services.

[9] Slade M, Amering M, Farkas M, Hamilton B, O’Hagan M, Panther G (2014). Uses and abuses of recovery: Implementing recovery-oriented practices in mental health systems. World Psychiatry.

[10] Adeponle AB, Whitley R, Kirmayer LJ, Rudnick A (2012). Cultural contexts and constructions of recovery. Recovery of People with Mental Illness: Philosophical and Related Perspectives.

[11] Slade M, Leamy M, Bacon F, Janosik M, Le Boutillier C, Williams J, Bird V (2012). International differences in understanding recovery: Systematic review. Epidemiology and Psychiatric Sciences.

[12] Davidson L, Rowe M, Tondora J, O’Connell MJ, Lawless MS: A Practical Guide to Recovery-Oriented Practice: Tools for Transforming Mental Health Care. Oxford, Oxford University Press, 2008.

[13] Wciórka J, Świtaj P, Anczewska M (2015). The stages of recovery in relation to the other subjective and objective aspects of psychosis. Psychiatry Research.

[14] Davidson L, Ridgway P, Kidd S, Topor A, Borg M (2008). Using qualitative research to inform mental health policy. The Canadian Journal of Psychiatry.

[15] Vaughn S, Schumm J, Sinagub J (1996). Focus Group Interviews in Education and Psychology.

[16] Song Y, Liu D, Chen Y, He G (2014). Using focus groups to design a psychoeducation program for patients with schizophrenia and their family members. International Journal of Clinical and Experimental Medicine.

[17] Kvale S (1996). InterViews: An Introduction to Qualitative Research Interviewing.

[18] Barbour R (2008). Doing Focus Groups.

[19] Kitzinger J, Barbour R, Barbour RS, Kitzinger J (1999). Introduction: The Challenge and Promise of Focus Groups. Developing Focus Group Research Politics, Theory and Practice.

[20] Brown JB, Crabtree BF, Miller WL (1999). The use of focus groups in clinical research. Doing Qualitative Research.

[21] Braun V, Clarke V (2006). Using thematic analysis in psychology. Qualitative Research in Psychology.

[22] Guest G, Bunce A, Johnson L (2006). How many interviews are enough? An experiment with data saturation and variability. Field Methods.

[23] Morse JM (1995). The significance of saturation. Qualitative Health Research.

[24] SAMHSA Issues Consensus Statement on Mental Health Recovery. 2006. https://www.power2u.org/downloads/SAMHSA%20Recovery%20Statement.pdf. Accessed 15 Feb 2016.

[25] Rapp CA, Goscha R: The Strengths Model: Case Management with People with Psychiatric Disabilities, 2nd edn, Oxford, Oxford University Press, 2006.

[26] Griffith JL, Myers N, Compton MT (2015). How can community religious groups aid recovery for individuals with psychotic illnesses?. Community Mental Health Journal.

[27] Hartley S, McArthur M, Coenen M, Cabello M, Covelli V, Roszczynska-Michta J (2014). Narratives reflecting the lived experiences of people with brain disorders: common psychosocial difficulties and determinants. PLoS One.

[28] Kwiatkowska A, Roszak J, Sikora R, Kuo BC, Karpinskij K, Gushchina T (2014). Kultura a strategie radzenia sobie ze stresem. Badania międzykulturowe. Psychologia Społeczna.

[29] Glover H, Ryan P, Ramon S, Greacen T (2012). Recovery, Lifelong Learning, Empowerment & Social Inclusion: Is a New Paradigm Emerging?. Empowerment, Lifelong Learning and Recovery in Mental Health: Towards a New Paradigm.

[30] Grant PM, Huh GA, Perivoliotis D, Stolar NM, Beck AT (2012). Randomized trial to evaluate the efficacy of cognitive therapy for low-functioning patients with schizophrenia. Archives of General Psychiatry.

[31] Laithwaite H, O’Hanlon M, Collins P, Doyle P, Abraham L, Porter S (2009). Recovery after psychosis (RAP): A compassion focused programme for individuals residing in high security settings. Behavioural and Cognitive Psychotherapy.

[32] Onken SJ, Craig CM, Ridgway P, Ralph RO, Cook JA (2007). An analysis of the definitions and elements of recovery: A review of the literature. Psychiatric Rehabilitation Journal.

[33] Sagan A, Panteli D, Borkowski W, Dmowski M, Domański F, Czyżewski M, et al: Poland: Health system review. Health systems in transition. 2011. http://www.euro.who.int/__data/assets/pdf_file/0018/163053/e96443.pdf. Accessed 15 Feb 2016. 22551527

[34] Green CA, Estroff SE, Yarborough BJH, Spofford M, Solloway MR, Kitson RS (2014). Directions for future patient-centered and comparative effectiveness research for people with serious mental illness in a learning mental health care system. Schizophr Bull.

[35] Söderström J: Focus Groups. Safety in Numbers? In: Hoglund K, Oberg M (Eds) Understanding Peace Research: Methods and Challenges. London and New York, Routledge, pp.146–164, 2011.

